# In silico analysis for determining the deleterious nonsynonymous single nucleotide polymorphisms of *BRCA *genes

**DOI:** 10.22099/mbrc.2019.34198.1420

**Published:** 2019-12

**Authors:** Fatemeh Yadegari, Keivan Majidzadeh

**Affiliations:** Genetics Department, Breast Cancer Research Center, Motamed Cancer Institute, ACECR, Tehran, Iran

**Keywords:** BRCA genes, SNPs, evolutionary analysis, computational tools

## Abstract

Recent advances in DNA sequencing techniques have led to an increase in the identification of single nucleotide polymorphisms (SNPs) in *BRCA1* and *BRCA2* genes, but no further information regarding the deleterious probability of many of them is available (Variants of Unknown Significance/VUS). As a result, in the current study, different sequence- and structure-based computational tools including SIFT, PolyPhen2, PANTHER, SNPs&GO, FATHMM, SNAP, PhD-SNP, Align-GVGD, and I-Mutant were utilized for determining how resulted BRCA protein is affected by corresponding missense mutations. FoldX was used to estimate mutational effects on the structural *stability* of BRCA proteins. Variants were considered extremely deleterious only when all tools predicted them to be deleterious. A total of 10 VUSs in *BRCA1* (Cys39Ser, Cys64Gly, Phe861Cys, Arg1699Pro, Trp1718Cys, Phe1761Ser, Gly1788Asp, Val1804Gly, Trp1837Gly, and Trp1837Cys) and 12 in *BRCA2* (Leu2510Pro, Asp2611Gly, Tyr2660Asp, Leu2686Pro, Leu2688Pro, Tyr2726Cys, Leu2792Pro, Gly2812Glu, Gly2813Glu, Arg2842Cys, Asp3073Gly, and Gly3076Val) were considered as extremely deleterious. Results suggested that deleterious *variants were mostly enriched in the* N- and C-terminal domain of the *BRCA1 *and *BRCA2* C-terminus. Utilizing evolutionary conservation analysis, we demonstrated that the majority of deleterious SNPs ensue in highly conserved regions of *BRCA* genes. Furthermore, utilizing FoldX, we demonstrated that alterations in the function of proteins are not always together with stability alterations.

## INTRODUCTION

Germ-line mutations in *BRCA1* and *BRCA2* tumor suppressor genes comprise most of the familial breast and ovarian cancer cases and significantly increase the chance of cancer development in carriers [1, 2]. *BRCA1* is responsible for encoding BRCA1 protein which consists of 1,863 amino acids, an N-terminal RING domain which binds with BARD1 to form a heterodimeric E3 ubiquitin ligase [3], a domain in the middle of the moiety interacting with DNA repair protein RAD51 [4], and a C-terminus containing two conserved BRCA1 C-terminal (BRCT) domains that mostly involve in tumor suppression, growth inhibition and transcription activation [5-7]. RING finger and BRCT domains are the most conserved regions of BRCA1 and mutations in these domains are in close association with hereditary breast and ovarian cancer development [8-10]. *BRCA2* is composed of 27 exons, which together encode a 3,418 amino acid protein referred to as BRCA2. The N-terminal domain of BRCA2 comprises of a transcription activating domain (residues18-105), while the middle region contains eight conserved BRC-repeated motifs [11], essential for binding with RAD51 and initiation of DNA repair [12]. A conserved DNA-binding domain has also been located in the C-terminal region of the BRCA2 protein which interacts with several other proteins, including DSS1 [13]. DSS1 is a highly conserved 70-amino-acid acidic protein involved in the repair of DNA double-strand breaks (DSB). So far, more than 1,781 mutations for *BRCA1* and 2000 mutations for *BRCA2* genes, including missense and insertion/deletion polymorphisms, etc. have been documented at the Breast Cancer Information Core (BIC) database. Most clinically relevant alterations detected in the *BRCA* genes are either nonsense or frameshift mutations, resulting in truncation or inactivation of the protein. These variants significantly deplete proteins function, early detection of them can contribute to prompt initiation of therapy or planning proper therapeutic strategies for preventing or delaying future cancer occurrence. Contrarily, in most cases, genetic variants, including missense and silent substitutions plus alterations in intronic and regulatory regions, provide no specific information regarding the function of the altered protein and generally referred to as variants of uncertain significance (VUS). Existing experimental methods for identifying the role of VUSs is too costly and time-consuming. Hence, the development of a low-cost and fast method for interpreting VUSs is valuable. Application of computational approaches for discriminating deleterious nonsynonymous substitution SNPs (nsSNPs) from neutral ones has emerged as an ideal strategy for exploring the mutation-structure-function relationship. Recently, several attempts have been dedicated to developing an improved computational approach for detecting deleterious mutations [14, 15]. In the current study, the deleterious effects of mutations were predicted by several computational tools with different features, including evolutionary conservation, structural information, and biophysical characterization.

## MATERIALS AND METHODS

**SNP information Retrieval: **SNPs in *BRCA1* and *BRCA2 *coding regions were retrieved from the BIC database (https://research.nhgri.nih.gov/bic/). Furthermore, data regarding the association between variations and their further disease-causing potency were obtained from the International Agency for Research on Cancer (IARC) [16]. The IARC database is a panel of experts that classified variants based on segregation data, prediction tools, and co-occurrence with a pathogenic BRCA variant, and so on. According to the IARC database, the variants were categorized into five classes as follows: 1) pathogenic/Class 5, 2) likely pathogenic/Class 4, 3) variant of uncertain significance/Class 3 and 4) likely benign/Class 2 and 5) benign/Class 1. 

**Prediction based on sequence homology:** In this study, different sequence- and structure-based computational tools including, SIFT [17], PolyPhen 2 [18], PhD-SNP [19], FATHMM [20], PANTHER [21], SNAP [22] and SNPs&GO [23] were used for determining the functional significance of nsSNPs in *BRCA* genes. A summary of the methods is presented in supplement file Table 1.

**Prediction based on biophysical characterization:** Align-GVGD is a program for combining protein multiple sequence alignments (MSA) and biophysical characteristics of amino acids for precise predicting that whether a missense substitution is deleterious or neutral. The Grantham Variation (GV) score calculates the grade of biochemical variation among amino acids presented at a particular position in the MSA, and the Grantham Deviation (GD) imitates the biochemical distance between the mutant and correct amino acid in its prime position. Based on Align-GVGD, missense substitutions categorize into seven grades (C0 (most likely neutral), C15, C25, C35, C45, C55, and C65 (most likely deleterious)) [24, 25]. The C45, C55, and C65 classes show that mutations affecting the protein function while the intermediate class (C35), and C0, C15, and C25 classes do not seem to have a functional impact.

**Prediction of protein stability with I-Mutant 3.0: **I-Mutant 3.0 is an SVM based protein stability prediction tool that estimates changes in protein stability upon single-point mutation in protein sequence or structure. Predicted free energy change value (DDG) is calculated from the changes between the Gibbs free energy (kcal/mol) of folded native proteins and unfolded mutant protein. Based on the calculated free energy changes, the software classifies predictions in three classes including neutral mutation (-0.5 ≤ DDG ≤ 0.5 kcal/mol), a large decrease (<-0.5 kcal/mol) and a large increase (> 0.5 kcal/mol) [26].

**Evolutionary conservation analysis: **The ConSurf server estimates the degree of conservation of each position, based on amino acid sequence comparisons. [27]. ConSurf scores range from 1 to 9: 1 indicates that the site is evolving rapidly (variable); 5 is the average, and 9 is *slowly evolving.*

**Statistical analysis: **Predictions of eight computational methods qualities including, SIFT[17], PolyPhen2 [18], PhD-SNP [19], FATHMM [20], PANTHER [21], SNAP [22], SNPs&GO [23], and I-Mutant 3.0 [26] were described by several statistical parameters in terms of sensitivity, specificity and MCC scores*. To achieve this* goal, the predictions made using computational tools were compared with the clinical *classification of variants* by the IARC database. The MCC is a correlation coefficient between the observed and predicted classifications, and varies between -1 and 1. An MCC coefficient of +1 represents the best possible prediction, whereas the MCC coefficient of -1 is regarded as the worst possible prediction. An MCC coefficient of 0 indicates a completely random prediction. Sensitivity, specificity, and MCC are calculated according to the following formulas:

$\mathrm{Sensitivity}=\frac{\mathrm{Tp}}{\mathrm{Tp}+\mathrm{Fn}}$

$\mathrm{Specificity}=\frac{\mathrm{Tn}}{\mathrm{Tn}+\mathrm{Fp}}$

$\mathrm{MCC}=\frac{\mathrm{Tp}\times \mathrm{Tn}-\mathrm{Fp}\times \mathrm{Fn}}{\sqrt[{2}]{\left(\mathrm{Tp}+\mathrm{Fp}\right)\left(\mathrm{Tp}+\mathrm{Fn}\right)\left(\mathrm{Tn}+\mathrm{Fp}\right)(\mathrm{Tn}+\mathrm{Fn})}}$

Where Tp,Tn, Fp and Fn are True positive, True negative, False positive and False negative, respectively.

**Homology modeling of human BRCA2-DSS1: **So far, homology modeling is the most accurate developed technique for constructing a reliable protein model based on amino acid sequences and the available crystal structure of homologous protein templates. In this approach, the quality of the constructed protein model mostly depends on template selection and the accuracy of sequence-template alignment. Currently, there is no crystal structure data for the human BRCA2 protein. Therefore, The human BRCA2 in complex with DSS1 was built using mouse BRCA2/DSS1 (PDB ID:1MIU) as the template with 76% sequence identity and 85% sequence similarity [13]. Of the 50 models generated with Modeller version 9.12 [28], the best *one* was *chosen* based on the *lowest DOPE score*. The quality of the *model *was evaluated using PROCHECK [29, 30] and *also* the structural *comparison* concerning the *template.*

**Analysis of amino acid substitutions on protein structural stability by FoldX: **FoldX plugin version 3.0 beta 6 for the YASARA program [31, 32] was utilized for calculating the impact of mutations on protein stability. FoldX is an empirical force field, calibrated by analyzing a collection of more than 1000 point mutations from 82 protein-protein complexes. ΔΔG was defined as the free energy difference between the wild-type ΔG(WT) and mutant ΔG(MT) and estimates whether a mutation is stabilizing (ΔΔG<0) or destabilizing (ΔΔG>0). It is calculated according to the following formula: ΔΔG = ΔG(MT) – ΔG(WT)

## RESULTS

**Figure 1:**
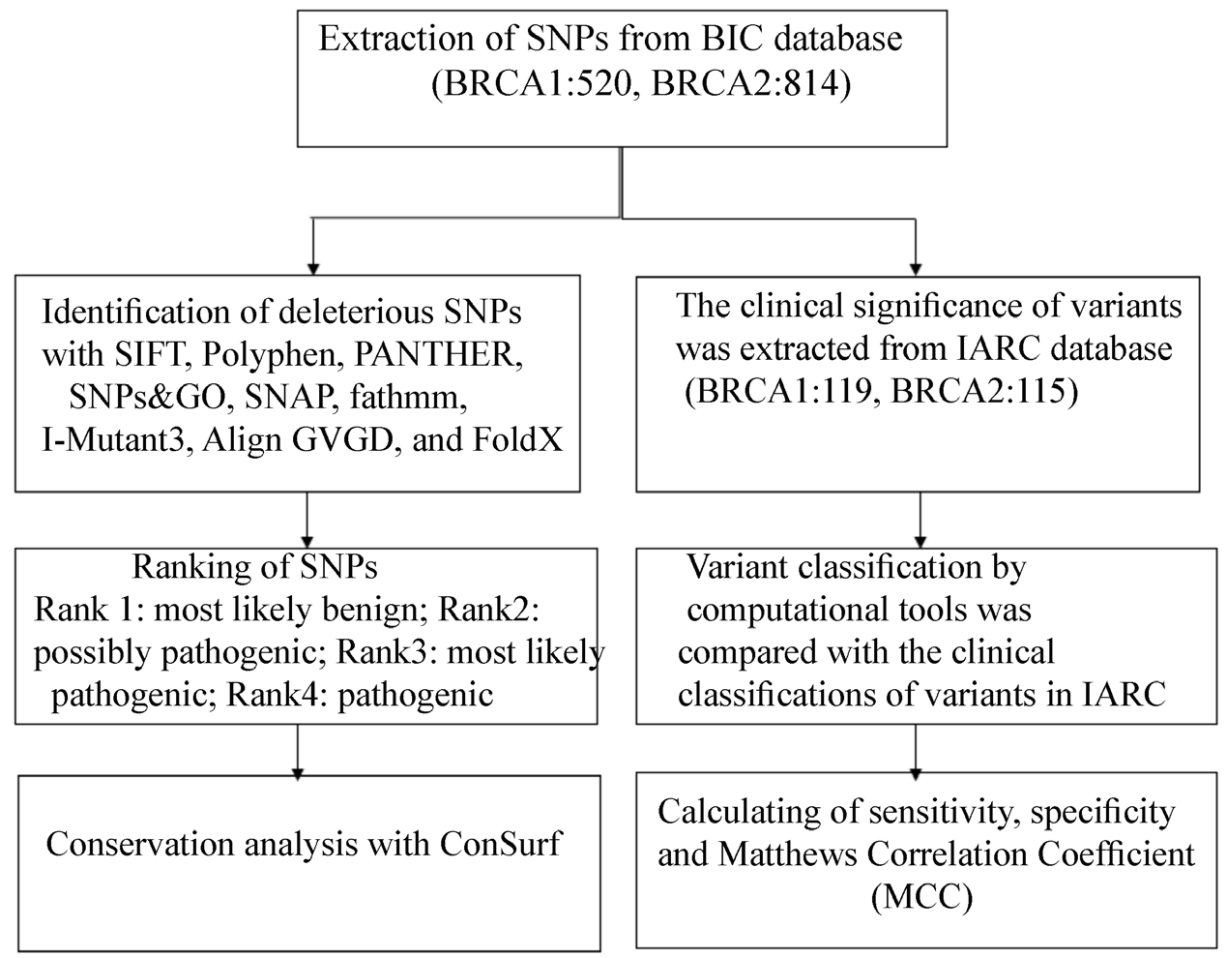
Flow chart for computational analysis of SNPs in this study

SNP data consisting of 520 missense mutations for *BRCA1* and 814 for human *BRCA2* genes were retrieved and considered for further computational analysis. These SNPs were considered for computational analysis. *The preformed steps* in this study are *summarized in *Figure 1*.*

**Figure 2:**
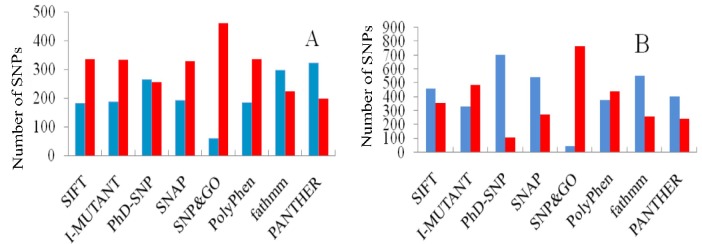
Distribution of predicted nsSNPs in * BRCA1* (A) and * BRCA2* (B) genes.

Figure 2 shows the distribution of the predicted deleterious and neutral variations in the human *BRCA *genes. 337 (64.8%), 335 (64.4%), 198 (38%), 327 (62.8%), 459 (88.2%), 254 (48.8%) and 223 (42.8%) out of 520 predicted missenses for *BRCA1* were classified as deleterious by SIFT, PolyPhen2, PANTHER, SNAP, SNPs&GO, PhD-SNP and FATHMM, respectively. Similarly, for *BRCA2*, SIFT, PolyPhen2, PANTHER, SNAP, SNPs&GO, PhD-SNP and FATHMM predicted 356 (43.7%), 438(53.8%), 242 (37.6%), 273 (33.5%), 766 (94.1%), 111 (13.6%) and 260 (31.9%) nsSNPs as deleterious, respectively (Fig. 2).

Align-GVGD was used to assign classes of *each *variant from C0 (most *likely neutral*) to C65 (most *likely deleterious*). For *BRCA2* variants, a great majority (683 of 814, 83.92%) fell into C0 class. Remaining SNPs were then classified as class 15 (n=28), class 25 (n=13), class 35 (n=16), class 45 (n=5), class 55 (n=14) and class 65 (n=55). For *BRCA1*, the majority of variants (n=413) fell into C0 class, and the remaining were classified as class 15 (n=16), class 25 (n=13), class 35 (n=11), class 45 (n=8), class 55 (n=8), and class 65 (n=51). In this study, substitutions with scores C45, C55 and C65 were considered as deleterious. The *list* of *all* *mutations with their prediction scores is *available in the S1 Appendix

I-Mutant 3.0 is an SVM-based tool, and it has been trained to predict protein stability changes upon single-point mutations, using structure and sequence information. The results for *BRCA2* indicated that 484 nsSNPs (59.4%) with negative ΔΔG values were less stable and deleterious, 316 SNPs (38.8%) were neutral, and 14 SNPs (1.7%) increased the stability of the protein. Similarly, for *BRCA1*, it gave an estimation of 333 stability-decreasing-nsSNPs (64%), while 182 SNPs (35%) were neutral to the mutation and 5 SNPs (0.96%) increased the stability of protein after mutation (S1 Appendix). 

The performance of computational tool results was assessed by calculating sensitivity, specificity, and MCC using registered variants in the IARC database as the gold standard. Experimental data regarding the clinical significance of 117 *BRCA1* variants and 115 *BRCA2* variants were obtained from the IARC database. These *mutations* were *shown* in the S1 *Appendix (column P)*. Of the seven computational approaches, PhD-SNP, SNAP, and SNPs&GO performed with a sensitivity score of 100% for *BRCA1*. Nevertheless, FATHMM (36.36%) performed the worst regarding sensitivity. PANTHER performed the best (68.48%, 0.5085) in terms of specificity and MCC. SNP&GO (17.39%) and SNAP (31.53%) performed worst in terms of specificity. On the other hand, FATHMM (-0.057) had the worst performance in terms of MCC (Table 1). For *BRCA2*, SN&GO, PolyPhen2, and FATHMM showed a sensitivity of 100%. Additionally, PhD-SNP (86.95%, 0.493) and FATHMM (70.65%, 0.466) performed the best in terms of specificity and MCC. However, SNP&GO was the worst in terms of specificity and MCC (2.17%, 0.05), while SNAP and I-Mutant in terms of sensitivity (66.6%) (Table 1).

**Table 1:** Statistical evaluation of various computational methods

		**SIFT**	**PolyPhen**	**PANTHER**	**PhD-SNP**	**SNP&GO**	**SNAP**	**FATHMM**	**I-Mutant**
***BRCA1***	**Tp**	21	20	21	22	22	22	8	14
	**Tn**	40	36	63	54	16	29	52	30
	**Fp**	52	56	29	38	76	63	40	62
	**Fn**	1	2	1	0	0	0	14	8
	**Sensitivity (%)**	95.45	90.91	95.45	100	100	100	36.36	63.64
	**Specificity (%)**	43.48	39.13	68.48	58.7	17.39	31.52	56.5	32.61
	**MCC**	0.32	0.2515	0.5085	0.4639	0.197	0.2856	-0.0569	-0.0314
***BRCA2***	**Tp**	11	12	9	9	12	8	12	8
	**Tn**	42	32	41	80	2	56	65	42
	**Fp**	50	60	27	12	90	36	27	50
	**Fn**	1	0	3	3	0	4	0	4
	**Sensitivity (%)**	91.6	100	75	75	100	66.67	100	66.67
	**Specificity (%)**	45.65	34.7	60.29	86.95	2.17	60.8	70.65	45.65
	**MCC**	0.2421	0.24	0.253	0.493	0.05	0.178	0.466	0.079

Tp: True positive, Tn: True negative, Fp: False positive, Fn: False negative

The accuracy of computational tools was improved by combining results from multiple tools [33]. So, we have used a ranking strategy to prioritize nsSNPs based on deleterious scores obtained from the computational prediction methods, including SIFT, PhD-SNP, PolyPhen2, SNPs&GO, SNAP, FATHMM and, I-Mutant 3.0. PANTHER was not able to predict the scores for the number of nsSNPs. The ranking scheme for *prioritizing of* mutations in this study is as follows: Variants predicted to be deleterious by zero or one of the seven tools were categorized as rank1, variants predicted to be deleterious by two or three of the seven tools were categorized as rank2, variants predicted to be deleterious by four or five of the seven tools were categorized as rank3, and rank4 variants predicted to be deleterious by six or seven tools. Mutations with ranking scores 3 and 4 were considered to be deleterious. Computational predictions for each SNP along with their ranking score are shown in the S1 Appendix.

The *variant was classified* to be extremely deleterious if it *was predicted *as *deleterious* by *all* the computational *tools, including* SIFT, PhD-SNP, PolyPhen2, SNPs&GO, SNAP, FATHMM, I-Mutant 3.0, and Align-GVGD. A total of 14 and 19 SNPs in *BRCA1 *and *BRCA2*, respectively, were categorized to be extremely deleterious. Among these mutations, 10 (Cys39Ser, Cys64Gly, Phe861Cys, Arg1699Pro, Trp1718Cys, Phe1761Ser, Gly1788Asp, Val1804Gly, Trp1837Gly, and Trp1837Cys) and 12 (Leu2510Pro, Asp2611Gly, Tyr2660Asp, Leu2686Pro, Leu2688Pro, Tyr2726Cys, Leu2792Pro, Gly2812Glu, Gly2813Glu, Arg2842Cys, Asp3073Gly and Gly3076Val) were VUSs in *BRCA1* and *BRCA2*, respectively. These SNPs can seriously disrupt the structural and functional features of BRCA proteins.

Amino acids participating in important biological processes, especially those located in enzyme-active sites or involved in protein-protein interactions, tend to be more evolutionarily conserved compared to the other residues. Therefore, mutations occurring at evolutionarily conserved sites are thought to be more deleterious compared to the ones at non-conserved positions. For this reason, we focused on substitutions predicted to be deleterious (mutations rank 3 and rank 4). Based on results obtained from the ConSurf server [27], most of the predicted deleterious mutations showed a significantly higher evolutionary conservation compared to the neutral ones (S1 Appendix).

**Figure 3:**
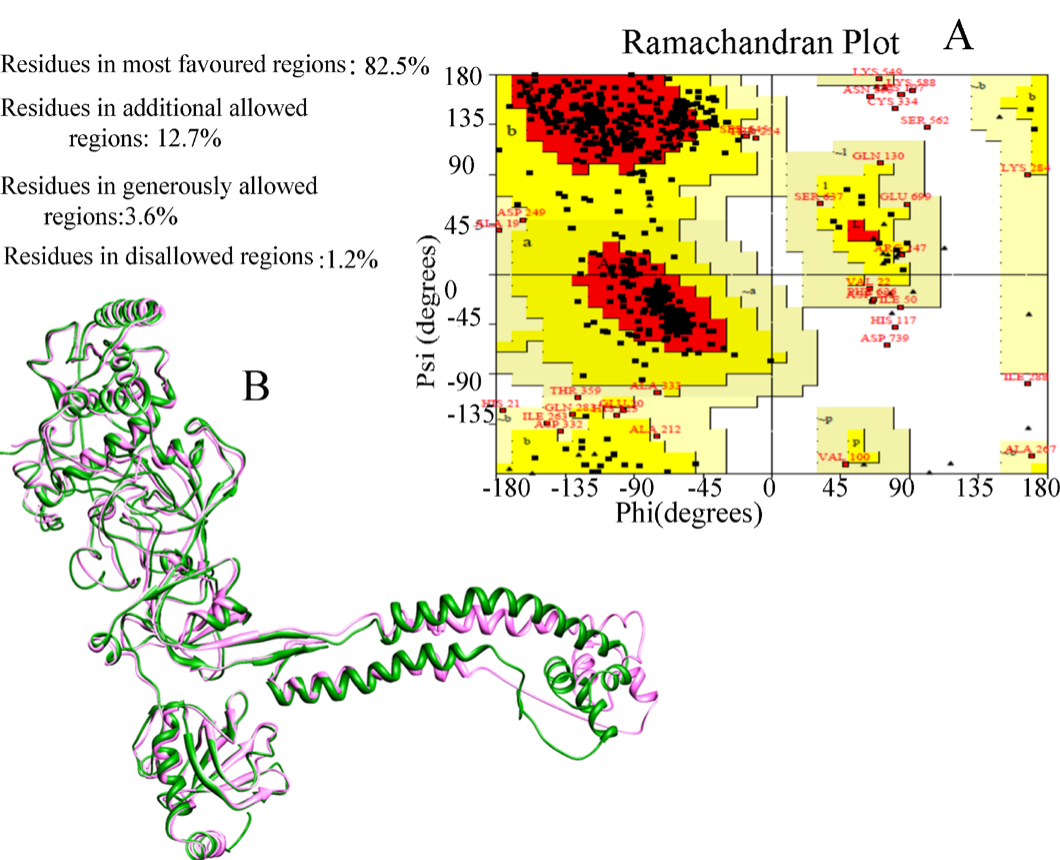
The quality assessment of the model generated with modeller. A) Ramachandran plot analysis of modeled BRCA2 structure generated by PROCHECK B) Comparison of model (pink) and crystal structure of mouse BRCA2 (green)

The human BRCA2 - DSS1 model was generated using modeller, and the best model was selected based on the lowest DOPE score. The *superimposition* of the template crystal structure of the BRCA2/DSS1 with predicted structure showed backbone RMSD 0.862Å. *Ramachandran plot* generated by *PROCHECK* *[29, 30] showed* that 82.5 %, 12.7% and 3.6% of the residues are in most favoured, allowed, and generously allowed regions, respectively (Fig. 3). These results showed that the obtained 3D model of BRCA2 is relatively satisfactory.

For stability analysis by FoldX, the human BRCA2*-*DSS1 model and the 3D structures of the BRCT repeat region, and ring domain of BRCA1 protein (PDB code: 1JNX and 1JM7) were used [34, 35]. At first, stability analyses with FoldX were restricted to *mutations* in which the atomic coordinates and the related *experimental data* were available in the IARC database (BRCA*1*=35, BRCA2=30, total=65). In this analysis, *mutations with a *free *energy change* greater than +3.0 kcal/mol *were considered* to be significant, based on previous experimentally-tested designs [31, 32]. The applied criteria (ΔΔG>3 kcal/mol) for the stability analysis of variants in the present study possessed 96% specificity, 48% sensitivity [36].

**Figure 4:**
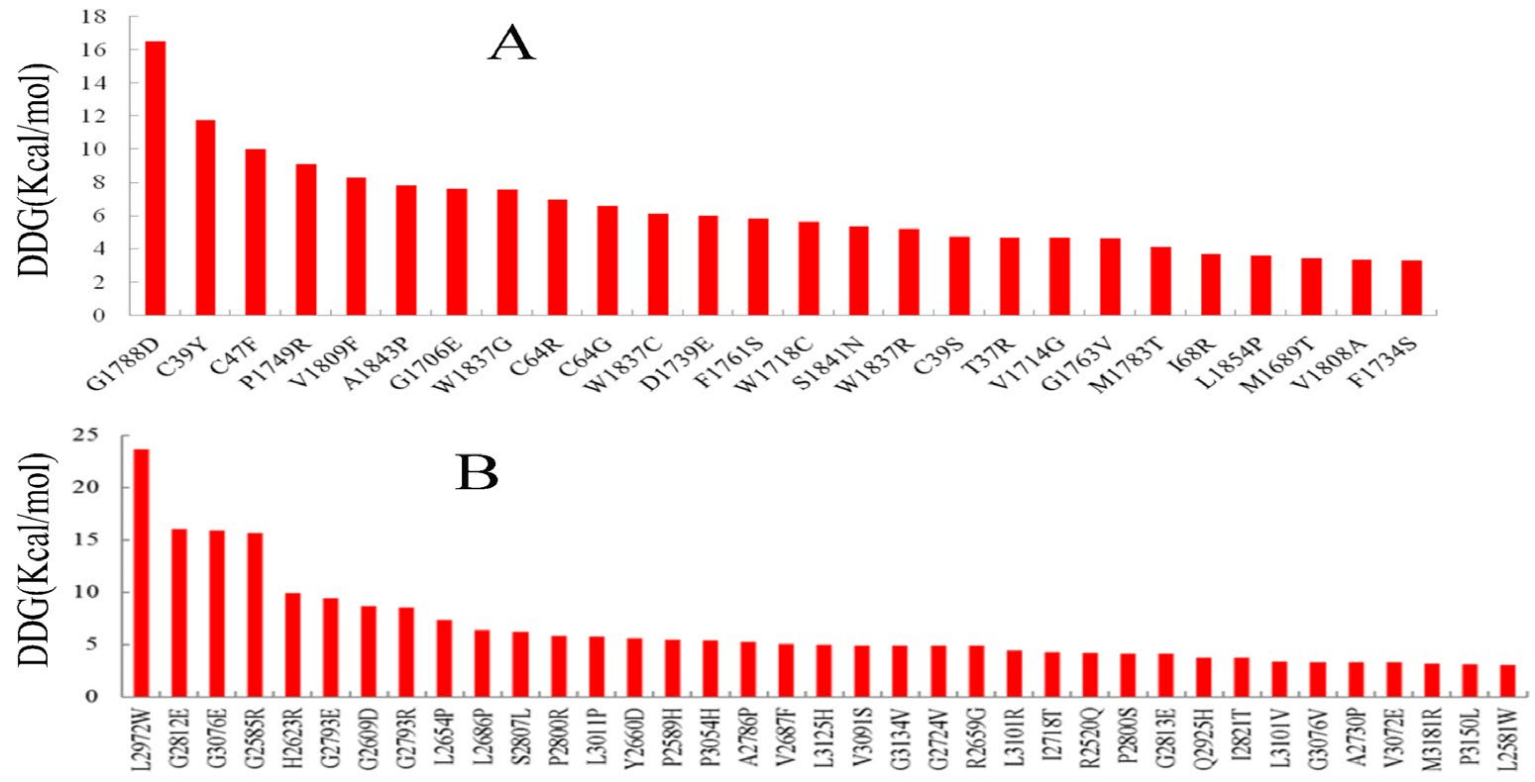
VUSs are predicted to destabilize the tertiary structure of *BRCA1* (A) and *BRCA2* (B) and so, considered deleterious.

Consequently, in the next step, we analyzed mutations in *BRCA1* N- and C-terminus and *BRCA2* C-terminus with unknown clinical importance. As depicted in figure 4, mutations with free energy changes greater than +3.0 kcal/mol are pathogenic. These data suggest that these mutations may play major roles in driving the *pathogenic related* states of proteins (Fig. 4 and S1 Appendix).

## DISCUSSION

SNPs play an important role in understanding the genetic basis of many multifaceted human disorders. However, the identification of functional SNPs remains a great challenge. Consequently, here, we performed an in silico analysis with the purpose of discrimination of pathogenic mutations from neutral ones. Different widely-used evolutionary-based methods including SIFT [17], PolyPhen2 [18], PhD-SNP [19], SNAP [22], SNPs&GO [23], PANTHER [21], I-Mutant [26], FATHMM[20] and Align-GVGD [24, 25] were employed for determining the functional significance of nsSNPs. Stehr et al. demonstrated that destabilizing mutations in tumor suppressor genes, which preferably take part in the core domains of proteins are frequently correlated with cancer [37]. Accordingly, to quantify the destabilization effect of mutations on *BRCA1/2* tumor suppressor genes, we computed the protein stability change upon these mutations utilizing I-Mutant [19] and the empirical forceﬁeld FoldX [31, 32]. In the FoldX approach, mutations with a ΔΔG>3 kcal/mol were considered highly destabilizing [36]. Comparing FoldX predictions with existing experimental data in the IARC database demonstrated *approximately* 96% specificity and 48% sensitivity. Although it has been demonstrated that FoldX is currently one of the best methods for calculating stability changes upon mutation, the method could not identify all potentially harmful mutations. The results presented in this study showed that alterations in protein function due to mutations do not always correlate to alterations in stability. For instance, the BRCA1 Cys1787Ser variant was classified as class 5 (definitely pathogenic) based on IARC classification criteria [38]. However, in this study, as well as previous research [39], this mutation has no serious unstabilizing effects on protein stability. Cancer-predisposing *mutations* that destabilize the BRCA1 structure likely to abolish the ability of BRCA1 for *transcriptional activation* *[*40*].* Val1714Gly mutation *abolishes the transcriptional activation* by BRCA1 in yeast and mammalian cells [41, 42]. *The* *results of this study showed* that at least part of the function defect associated with this mutation is likely due to protein destabilization. It has been shown that BRCA1 Gly1788Val mutation is associated with a significant decrease in transcription activation assay. However, NMR spectroscopy results indicate that this residue is essential for the stability of the BRCT domain of BRCA1 [43]. This result is also consistent with our finding that Gly1788Val strongly destabilizes the wild-type BRCA1 protein. The evolutionary analysis also demonstrated that mutations in the conserved region often lead to instability and function impairment. This trend is in agreement with previous studies, which found that pathogenic mutations occur more frequently in the conserved region [14, 44-46].

## Supplementary Materials

Supplement
