# Supplementary material for: In silico analysis for determining the deleterious nonsynonymous single nucleotide polymorphisms of BRCA genes
Source: Mol Biol Res Commun. 2019 Dec;8(4):141–50. doi: 10.22099/mbrc.2019.34198.1420 (PMC6995337; doi:10.22099/mbrc.2019.34198.1420)
Supplement: Supplement [file mbrc-8-141-s001.pdf]

Table 1: Summary of the prediction tools used in this study

Name	Type	Deleterious	Reference
**SIFT**	Conservation	<0.05	[17]
**PolyPhen**	Conservation, Protein structure, Protein sequence annotation	>0.15	[18]
**SNAP**	Conservation, Structural annotation	Non-neutral	[22]
**SNPs&GO**	Protein sequence, Functional annotation	>0.5	[23]
**PANTHER**	Protein sequence	<-3	[21]
**PhD-SNP**	Protein sequence		[19]
**FATHMM**	Conservation, protein sequence annotation	<-0.75	[20]
**I-Mutant**	Sequence	<-0.5	[26]
